# A Review of Circulating Tumour Cell Enrichment Technologies

**DOI:** 10.3390/cancers13050970

**Published:** 2021-02-26

**Authors:** Amelia J. Rushton, Georgios Nteliopoulos, Jacqueline A. Shaw, R. Charles Coombes

**Affiliations:** 1 Department of Surgery and Cancer, Imperial College London, Hammersmith Hospital, London W12 0NN, UK; georgios.nteliopoulos04@imperial.ac.uk (G.N.); c.coombes@imperial.ac.uk (R.C.C.); 2 Leicester Cancer Research Centre, University of Leicester, Leicester LE2 7LX, UK; js39@leicester.ac.uk

**Keywords:** circulating tumour cell (CTC), cancer, metastasis, liquid biopsy

## Abstract

**Simple Summary:**

Circulating tumour cells (CTCs) are cancer cells shed into the bloodstream from tumours and their analysis can provide important insights into cancer detection and monitoring, with the potential to direct personalised therapies for the patient. These CTCs are rare in the blood, which makes their detection and enrichment challenging and to date, only one technology (the CellSearch) has gained FDA approval for determining the prognosis of patients with advanced breast, prostate and colorectal cancers. Here, we review the wide range of enrichment technologies available to isolate CTCs from other blood components and highlight the important characteristics that new technologies should possess for routine clinical use.

**Abstract:**

Circulating tumour cells (CTCs) are the precursor cells for the formation of metastatic disease. With a simple blood draw, liquid biopsies enable the non-invasive sampling of CTCs from the blood, which have the potential to provide important insights into cancer detection and monitoring. Since gaining FDA approval in 2004, the CellSearch system has been used to determine the prognosis of patients with metastatic breast, prostate and colorectal cancers. This utilises the cell surface marker Epithelial Cell Adhesion Molecule (EpCAM), to enrich CTCs, and many other technologies have adopted this approach. More recently, the role of mesenchymal-like CTCs in metastasis formation has come to light. It has been suggested that these cells are more aggressive metastatic precursors than their epithelial counterparts; however, mesenchymal CTCs remain undetected by EpCAM-based enrichment methods. This has prompted the development of a variety of ‘label free’ enrichment technologies, which exploit the unique physical properties of CTCs (such as size and deformability) compared to other blood components. Here, we review a wide range of both immunocapture and label free CTC enrichment technologies, summarising the most significant advantages and disadvantages of each. We also highlight the important characteristics that technologies should possess for routine clinical use, since future developments could have important clinical implications, with the potential to direct personalised therapies for patients with cancer.

## 1. Introduction

**Figure 1:**
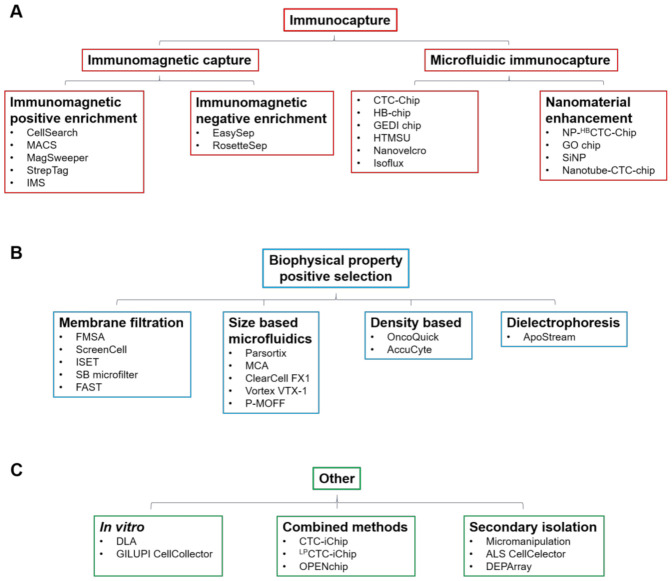
Summary of circulating tumour cell (CTC) enrichment technologies. (**A**) Immunocapture methods including immunomagnetic positive and negative enrichment methods, microfluidic immunocapture methods, nanomaterial immunocapture enhancement and their relevant technologies; (**B**) Biophysical property enrichment methods including membrane filtration, size-based microfluidics, density based and dielectrophoresis and associated technologies; (**C**) Other methods including in vitro, combined and secondary isolation methods and associated technologies.

Circulating tumour cells (CTCs) are shed into the bloodstream from both primary and metastatic tumours and those that are able to survive in the circulation represent metastatic precursor cells [1]. CTCs are important biomarkers for disease and are a powerful tool to study tumour progression and evolution. They represent a rare and heterogeneous population of cells, typically accounting for ∼1 cells for every 10^5^–10^6^ peripheral blood mononuclear cells (PBMCs), so a key challenge for their clinical utility is the development of standardised isolation and characterisation technologies [2]. There are numerous technologies that have been developed to enrich CTCs from normal hematopoietic cells that rely on physical and biological properties of CTCs, including size, density, cellular charge and expression of cellular markers. The enrichment techniques (Table 1) can broadly be divided into immunocapture methods that differentiate cells based on epithelial cell surface marker expression, notably epithelial cell adhesion molecule (EpCAM) (Figure 1A), and those that differentiate based on distinct biophysical properties (Figure 1B,C). If CTC enrichment and characterisation is to be routinely used in the clinical setting, technologies must ideally meet several criteria: they must have high detection and recovery rates, with accurate throughput sample processing and enumeration capability. Further, they must be generally fully automated and easy to use, with little to no pre-processing of blood required. Finally, if they are to have wide clinical applicability, they must be able to detect heterogeneous cells from a wide range of different cancers.

## 2. Immunomagnetic Positive Enrichment

Immunocapture methods selectively target markers present on CTCs for a positive enrichment approach and/or markers present on leukocytes for their depletion in a negative enrichment approach. The CellSearch (Menarini Silicon Biosystems, Bologna, Italy) is the ‘Gold Standard’ platform for CTC isolation and couples immunomagnetic enrichment using ferrofluid coated with anti-EpCAM antibodies with in-device immunostaining for the cancer cell marker cytokeratin (CK) -8, -18 and -19, the leukocyte marker CD45 and nuclear stain 4′,6-diamidino-2-phenylindole (DAPI) [52]. Recovered cells are imaged using the associated CellTracks Analyser, which images individual cells using four fluorescence channels and presents the final images to the operator for review and CTC enumeration. A CTC is defined as a CK-positive CD45-negative cell with an intact nucleus as evidenced by DAPI staining and must be of round or oval shape with a diameter of at least 4 µm [53]. Using the fourth channel of the CellTracks Analyser, an additional marker can be added such as a fluorescently labelled antibody (anti-HER81 mouse monoclonal antibody) for human epidermal growth factor 2 (HER2) expression in metastatic breast cancer [54]. The manufacturers claim recovery rates of ≥85% with clinical detection rates of 71.4%, but several groups have suggested figures lower than this, with recovery rates ranging from 42% to 90% and clinical detection rates between 20% and 77.5% [3,4,55,56,57,58]. The CellSearch instrument has been approved by the US Food and Drug Administration (FDA) for CTC enumeration in metastatic breast, colorectal and prostate cancers, where CTC enumeration has been used as a prognostic tool, since it has been associated with overall survival and/or progression free survival in those advanced cancers [5,59,60]. At the time of writing, a search of clinicaltrials.gov (accessed on 20 February 2021) reveals 55 clinical trials featuring the CellSearch, the majority of which are prostate and breast cancer trials however other cancer types include colorectal, lung, oesophageal, melanoma, head and neck, gastric, endometrial, renal and pleural neoplasms [59]. The CellSearch has many advantages such as ease of use, relatively quick processing time with the ability to process eight different blood samples in parallel, in-device staining and the addition of the CellTracks Analyser to quickly and efficiently allow for CTC enumeration [5,6]. There is also an option to recover live CTCs, whereby blood collected in EDTA tubes is processed using the CellSearch Epithelial Cell Profile Kit. Cells are captured and enriched based on EpCAM expression but are not fixed, permeabilised and stained [60]. The CellSearch platform was first approved by the FDA for the clinical detection of CTCs in peripheral blood in 2004 and to this date remains the only CTC enrichment technology to gain FDA approval. It is important to note that as the CellSearch is currently the only CTC enrichment technology to be used clinically, all other technologies detailed below are, at the time of writing, for research use only.

The major drawback of immunocapture methods, including the CellSearch, is the EpCAM bias placed upon the enriched CTC population. It is widely acknowledged that during intravasation, as part of the metastatic cascade, cancer cells undergo epithelial to mesenchymal transition (EMT), whereby they lose epithelial characteristics and gain a mesenchymal phenotype to facilitate escape into the blood stream [61]. Cells that have undergone EMT may exhibit partial or complete downregulation of epithelial markers EpCAM, E-cadherin and cytokeratins and an upregulation of mesenchymal-specific markers such as vimentin and N-cadherin, associated with increased expression of EMT-associated transcription factors Slug, Snail and Twist [62]. Such a downregulation of EpCAM would therefore render cells undetectable using such immunocapture methods. It has been reported that these ‘mesenchymal CTCs’ exhibit increased metastatic potential and are more aggressive metastatic precursors than their epithelial counterparts [63,64]. Therefore, CTC isolation based on EpCAM expression alone may give an inaccurate CTC count and also limit the heterogeneity of the CTC population for downstream analysis. It has also been reported that mesenchymal CTCs are more prevalent in patients not responding to therapy, with mesenchymal-like and stem cell-like CTCs found in 62% and 44% of patients not responding to treatment, compared to 10% and 5% in responders, respectively [65]. The CellSearch immunocapture method is therefore currently limited to epithelial type cancers and not applicable to non-epithelial cancers such as sarcomas, lymphomas and neural tumours which do not express EpCAM [7]. 

A number of alternative immunomagnetic capture methods are available that include MACS (Miltenyi Biotec; Bergisch Gladbach, Germany), MagSweeper, the Strep-tag system (IBA Lifesciences, Göttingen, Germany) and the biomimetic immunomagnetosome (IMS) which differ from the CellSearch in terms of their capture or release mechanisms. For CTC separation using the MACS kit, whole blood is firstly incubated with target antibody coated superparamagnetic beads, including antibodies against CK and HER2 as well as the most commonly used EpCAM. The blood is then passed through a column containing ferromagnetic steel wool fibres whilst subjected to a magnetic field, with each column able to process up to 15 mL blood. The CTC-bead conjugates are held within the steel wool fibres and are easily eluted upon removal of the magnetic field [8]. In addition to the separation columns, Miltenyi has also developed the autoMACS Pro Separator. This is a fully automated machine combining in-device labelling with MACS reagents and magnetic separation. The Pro Separatorcan process up to six samples per run with the option to reload the eluted positive fraction onto a second column for increased purity. However, reports have suggested poor reproducibility using the system with capture efficiencies ranging from 25% to 90% and have suggested the MACS system is better suited for tissue samples and that high yielding pure CTC samples are difficult to obtain from blood [9].

The MagSweeper adopts an alternative method of CTC capture using neodymium rods covered with nonadherent plastic sleeves. Whole blood is firstly incubated with magnetic beads coated with anti-EpCAM antibodies and then the robotically controlled neodymium rod sweeps through the blood in a 6 well plate to capture labelled CTCs [10]. The nonadherent plastic sleeves allow for multiple capture and release cycles to increase capture efficiency and multiple magnetic rods can be fabricated to the automated system to allow for the simultaneous processing of multiple samples. Generally, 60–70% capture efficiency has been reported but a wide range of sample purities have been reported ranging from 50% to 100%, however this variety may be linked to the number of capture rounds performed since multiple rounds of capture and release will result in greater capture efficiency but a lower purity recovery [10,11]. The device is quick at processing with 9 mL blood processed in 1 h and captured CTCs remain viable for downstream analysis [66]. 

One group combined MagSweeper isolation with mRNA-seq to assess any effect the enrichment process may have on cells during the isolation process [11]. They performed initial validation experiments using LNCaP cells before and after MagSweeper isolation and using Illumina sequencing, they were able to show the enrichment process has minimal effect on CTC transcriptomes. They then isolated CTCs from 13 metastatic prostate cancer patients using two rounds of MagSweeper isolation followed by micromanipulation to isolate 67 single CTCs without leukocyte contamination. Of the resulting CTCs, 21% had good quality RNA, 37% were partially degraded and 42% were found to be fully degraded. They observed the quality of CTC RNA to be somewhat patient specific, suggesting this may be a result of the therapy the patient was on at the time of blood sampling as opposed to any effect from the enrichment process itself. When sequencing the CTCs, they observed the upregulation of spindle-associated transcripts in CTCs from patients on taxane chemotherapy. Taxanes act by blocking cell cycle progression through the inhibition of microtubule dynamics, so it is possible this upregulation could represent a response or development of resistance to taxane chemotherapy, conferring possible clinical implications [67]. 

The Strep-tag system utilises the same immunomagnetic enrichment approach as the CellSearch, however with a different CTC release mechanism. Whole blood is incubated with magnetic beads coated with strep-tactin (strep-tactin-coated magnetic beads (STMBs)) which are then conjugated with strep-tag-II derived IgG [68]. Following magnetic enrichment, the simple addition of d-biotin competitively binds to the strep-tactin on the STMBs, allowing for easy release of CTCs [12]. Anti-EpCAM-IgG-STMBs showed a capture efficiency of 79% of cancer cell lines spiked into healthy blood, with a release efficiency of 70% upon addition of biotin, releasing CTCs with 85% viability [13]. In addition to the anti-EpCAM-IgG-STMBs, they also have beads conjugated to multiple antibodies including EpCAM/epidermal growth factor receptor (EGFR), EpCAM/HER2 and a cocktail of all three. The addition of multiple antibodies results in increased CTC capture (82–86%, cell line and antibody dependent) and broadens the diversity of cells recovered, allowing for a better representation of heterogeneity than using EpCAM alone.

The system has been successfully tested on cell lines from a variety of different cancer types including lung, gastric, oesophagus, pancreatic and liver [13]. A direct comparison was made with the CellSearch. Here, blood samples from five patients (four colorectal and one breast) were run in parallel on the two devices, with anti-EpCAM/EGFR/HER2-IgG-STMBs used for isolation using the Strep-tag system. Recovery was 12–72 CTCs in 1 mL blood and 0–23 CTCs in 7.5 mL blood from the Strep-tag system and CellSearch, respectively, concluding that their system results in higher recovery rates due to the advantageous use of multiple antibodies [13].

The IMS approach coats magnetic nanoclusters with leukocyte membrane fragments (the magnetosome) which in turn are coated with anti-EpCAM antibodies to form an immuno-magnetosome [14]. During magnetic enrichment, leukocytes are repelled by the immuno-magnetosome due to their homology, resulting in a higher purity enrichment up to an almost undetectable leukocyte background. They reported ~90% capture efficiency of CTCs from whole blood within 15 min with very high purity recoveries. However, this system has only been verified using cell lines and is yet to be tested on patient samples. The same group then went on to develop a magnetically controlled nickel pattern microfluidic device in combination with biomimetic magnetosomes, whereby the immuno-magnetosomes are arranged in a planar field on the chip with the idea that this would enhance interactions between CTCs and immune-magnetosomes for greater capture efficiency. They reported the same capture efficiency as previously (~90%), however capture using the microfluidic chip allowed for easier enumeration using confocal laser scanning microscopy, providing capture and enumeration within 20 min. The device is highly sensitive and could capture as little as five cells spiked into healthy blood, however this is still to be tested on patient samples [14]. 

Overall, the CellSearch platform remains a leader in the field of immunomagnetic CTC capture due to the automated in-device cell staining and the use of the CellTracks Analyser for enumeration. Some of the other technologies may outperform the CellSearch in terms of CTC capture efficiency, however post processing cell staining and manual enumeration is a timely process and therefore not applicable for routine clinical use. Several groups have reported low sensitivity of the CellSearch, with its inability to detect cells expressing lower levels of EpCAM. Blood spiking experiments carried out using cell lines expressing differing levels of EpCAM (MDA-MB-231, PC3 and SKBR3 cells; low, medium and high EpCAM expression, respectively) saw recovery rates of 12%, 40% and 90%, respectively [4]. The seeming lack of sensitivity of the CellSearch, along with its inability to detect mesenchymal-like CTCs suggests that other technologies adopting different isolation mechanisms may be better suited for the routine detection of CTCs from a wide range of cancer types, but as yet this needs better definition.

## 3. Immunomagnetic Negative Enrichment

Immunomagnetic negative enrichment utilises the same immunomagnetic technology, using magnetic beads coated with anti-leukocyte antibodies to deplete the leukocyte population [69]. Most commonly, anti-CD45 antibodies are conjugated to magnetic beads for leukocyte depletion. For example, the EasySep (Stemcell Technologies, Vancouver, BC, Canada) technology uses a magnetic field to retain leukocytes, while the resulting supernatant retains the heterogeneous population of label free CTCs since no antigen targeting is involved. Each sample takes 25 min to process, capturing label free, viable cells. However, CTC recovery is extremely variable, reported at 24% ± 19% by one group and 58% ± 8% by another [15,16]. When isolating CTCs from patients with a range of different epithelial type cancers, CTCs were detected in 56% (47 of 84) of patients, with a wide range of detection rates seen within different subtypes (44% colon, 50% ovarian, 80% gastric, 100% lung) [16].

The RosetteSep (Stemcell Technologies) utilises a multitude of antibodies targeting several blood cell markers (CD2, CD16, CD19, CD36, CD38, CD45, CD66b and glycophorin A). Upon mixing the tetrameric cocktail with the blood, a rosette network of ‘unwanted blood cells’ is formed and a subsequent Ficoll density centrifugation allows for the removal of leukocytes and red blood cells (RBCs) from the sample. A group optimising the RosetteSep Ficoll procedure obtained an average recovery of 62.5% when testing spiked human ovarian and prostate cancer cell lines in healthy blood. This group was also able to detect CTCs in blood samples from 90% (18/20) of metastatic epithelial ovarian cancer patients and 76.9% (10/13) of prostate cancer patients [17]. Another group carried out a direct comparison between the RosetteSep and CellSearch in 19 patients with Merkel Cell carcinoma (MCC), with CTCs detected in 42% and 26% of patients using the RosetteSep and CellSearch, respectively, with only 16% of patients positive using both methods [70].

The RosetteSep is currently being used alongside the ApoStream technology to isolate CTCs from pancreatic patients in a small phase I trial (NCT02349867) (Table 2); using a regimen of sorafenib and vorinostat with gemcitabine and radiation following chemotherapy, aiming to determine the doses and schedule of the concurrent chemoradiation combination.

Negative enrichment methods are advantageous as they allow for the separation of CTCs independent of any cell surface marker expression and therefore may have a wide clinical applicability. However, due to the rare nature of CTCs in the blood, the process of negative depletion often results in a relatively low recovery rate as CTCs may be trapped within a mass of blood cells and therefore included in the depleted cell fraction and inadvertently removed in the process. It has recently been described that CTCs occasionally couple with neutrophils within the bloodstream to increase the metastatic potential of CTCs and therefore represent an interesting group of cells for researchers to study [71]. In addition, one group characterised CTCs in pancreatic ductal adenocarcinoma (PDAC) patients and discovered a novel population of hybrid cells expressing both CTC and leukocyte markers which appear to have been formed from a fusion between macrophages and cancer cells termed ‘tumacrophages’ [72]. Both CTC-white blood cell (WBC) clusters and tumacrophages may represent important cells in the circulation and would be removed from the final enriched population of cells using negative enrichment methods.

**Table 2:** Clinical trials including CTC analysis using different CTC enrichment technologies (clinicaltrials.gov; accessed on 20 February 2021). Cancer type, study type and status, study start date and estimated or actual study completion date and estimated or actual number of patients enrolled are also shown.

Technology	Trial Identifier	Cancer Type	Status	Study Type	Study Start Date	Estimated/Actual Completion Date	Estimated/Actual Number of Patients Enrolled
RosetteSep	NCT02349867	Pancreatic	Active	Phase I	January 2015	September 2024	23
CTC-Chip	NCT00888134	Solid neoplasm	Completed *	Phase II	July 2009	January 2015	28
	NCT01961713	Prostate	Recruiting	Observational	April 2010	August 2022	200
	NCT01734915	Lung	Completed [73]	Observational	November 2012	September 2016	40
	NCT02812680	Oesophageal	Recruiting	Observational	June 2016	June 2021	200
	NCT02630615	Lung	Active	Observational	September 2017	August 2021	41
GEDI chip	NCT01718353	Prostate	Completed [74]	Phase II	March 2013	August 2015	63
Nanovelcro	NCT01834651	Prostate	Completed [75]	Phase II	April 2013	July 2016	17
FMSA	NCT01722903	Colorectal	Completed [76]	Observational	April 2012	June 2015	25
ScreenCell	NCT03797053	Melanoma	Unknown	Observational	April 2015	Junee 2019	450
	NCT02610764	Oesophageal	Completed *	Interventional	November 2015	December 2016	19
ISET	NCT00818558	Lung	Unknown	Interventional	October 2008	October 2012	520
	NCT01776385	Pleural neoplasms	Completed *	Interventional	February 2012	March 2018	9
	NCT02372448	Lung	Completed [77]	Interventional	January 2015	November 2019	206
	NCT02500693	Lung	Unknown	Interventional	October 2015	September 2019	600
	NCT02827344	Lung	Recruiting	Observational	October 2015	December 2021	200
	NCT02554448	Rectal	Unknown	Interventional	January 2016	December 2016	80
	NCT03328559	Bronchial	Active	Interventional	March 2016	December 2020	6
	NCT02979470	Colorectal	Unknown	Observational	September 2016	September 2019	100
	NCT04702633	Prostate	Not yet recruiting	Observational	February 2021	February 2024	200
Parsortix	NCT02781272	Ovarian	Active	Observational	June 2016	June 2022	200
	NCT02785731	Ovarian	Completed *	Observational	July 2016	July 2018	204
	NCT03771404	Lung	Recruiting	Interventional	January 2018	December 2021	50
	NCT03427450	Breast	Completed *	Observational	March 2018	December 2019	421
	NCT04021394	Prostate	Recruiting	Observational	June 2019	December 2025	40
ClearCell	NCT02370303	Lung	Completed *	Observational	August 2014	July 2016	23
	NCT04696744	Head and neck	Not yet recruiting	Observational	February 2021	January 2025	40
ApoStream	NCT02349867	Pancreatic	Active	Phase I	January 2015	September 2024	23
	NCT02466802	Solid neoplasm	Completed *	Phase I	July 2015	January 2019	32

(The CellSearch features in 55 clinical trials (clinicaltrials.gov; accessed on 20 February 2021)); * Results not yet published.

## 4. Microfluidic Immunocapture

Microfluidics involves the controlled flow of blood through a chip designed to capture CTCs either based on cell surface marker expression (label dependent immunocapture) or based on cell size (label independent, size-based enrichment) [78]. Several groups have combined immunocapture with microfluidics to flow blood at a controlled flow rate over anti-EpCAM coated walls or microposts for increased contact and therefore enhanced capture efficiency.

The CTC-Chip, developed by Massachussets General Hospital (MGH), contains 78,000 anti-EpCAM coated micropillars, providing a large surface area for CTC capture. The device uses a very slow flow rate of 1–2 mL blood per hour, however this results in high viability of released cells (98%), demonstrating the minimal sheer stress the cells experience when processed through the device [18]. The chip has been tested on lung, colorectal, pancreatic, breast and prostate cancers and successfully identified CTCs in 99% (115 of 116) patients with 50% purity [19]. The group performed cell line spiking experiments, yielding recovery rates of >60% and were also able to isolate CTCs from 7/7 (100%) patients with early-stage prostate cancer, where CTCs are often present only in small numbers, suggesting high sensitivity and specificity of the chip. The complex geometry of the chip, however, proved difficult to scale up for high throughput production and such a slow flow rate means the chip would not be viable for routine clinical use.

One group isolated CTCs using the CTC-Chip from 23 metastatic non-small cell lung carcinoma (NSCLC) patients with known EGFR mutant tumours who had all received treatment with tyrosine kinase inhibitors [79]. Tyrosine kinase inhibitors such as gefitinib and erlotinib are used to treat NSCLC patients with activating mutations in the EGFR gene. Unfortunately, due to the acquisition of a secondary EGFR mutation (T790M) which hinders drug binding, patients often become resistant and relapse within 1 year of treatment initiation [80]. Following CTC-Chip enrichment, CTCs were detected in all patients, suggesting high sensitivity of the device and CTCs from 20 of these patients yielded DNA of sufficient quality and quantity for further analysis. Using a selective androgen receptor modulator (SARMS) assay, they detected activating EGFR mutations in 95% (19 of 20) patients, with the EGFR T790M mutation identified in 55% of the patients. The group also analysed serial CTC samples from a subgroup of patients collected over the course of their treatment. They reported emergence and increased prevalence of the T790M mutation, conferring the acquisition of drug resistance and suggesting that a change in treatment to second line irreversible tyrosine kinase inhibitors (TKI) or combination targeted therapies may be beneficial for the patients.

The CTC-Chip is currently in use in several clinical trials (Table 2); to evaluate the relationship between CTC burden and pathologic stage of primary prostate cancer patients before and after prostatectomy (NCT01961713), to assess the use of CTCs and plasma micro RNA as biomarkers of cancer and predictive markers for neoadjuvant therapy in patients with oesophageal adenocarcinoma (NCT02812680) and to establish CTC derived xenografts from patients with lung cancer to assess novel DNA repair inhibitors for a personalised therapeutic approach (NCT02630615). The CTC-chip was also used to isolate CTCs from patients with EGFR mutant tumours in trial NCT01734915, where they detected the T790M mutation in CTCs and/or ctDNA in 35% of patients for whom the mutation was not detected in the tumour biopsy. They showed that a combination of CTC and ctDNA analysis was optimal for the detection of T790M in patients who had progressed on EGFR TKI therapy, highlighting the importance of this complementary approach for treatment decisions [73].

The CTC-Chip was the first of four generations of chips developed by MGH; superseded by the herringbone (HB)-chip, CTC-iChip and most recently the ^LP^CTC-iChip [20,49,50]. The CTC-iChip and ^LP^CTC-iChip employ combinatorial approaches for CTC enrichment and will be discussed later.

Their second-generation HB-chip consists of eight microchannels designed with herringbone (chevron) grooves on the upper surface of the chip with the inner walls of the channels functionalised with anti-EpCAM antibodies [20]. The herringbone grooves act to disrupt the laminar flow of the blood, increasing the number of interactions between CTCs and antibody coated surfaces within the chip [81]. This therefore negates the need for the complex micropost geometry as seen in the CTC-Chip, making the HB-chip easier to produce whilst not compromising on recovery rates.

The chip was validated using PC3 cells spiked into healthy blood and recovery rates from the HB-chip were compared with that of a traditional smooth roof chip [20]. PC3 cells were chosen due to their relatively low expression of EpCAM. The HB-chip has capture efficiencies of 79% ± 4.5% compared to the flat chamber with 29% ± 4.3%. A larger version of the HB-chip was then designed for use with larger volume clinical samples and capture efficiency increased to 91.8% ± 5.2% with spiked PC3 samples. Comparing purities of recovered samples with the two types of chip, the HB-chip saw 14.0% ± 0.1% compared to 9.2% ± 0.1% purity for the CTC-chip. The HB-chip could also recover cells with 95% ± 0.6% viability. When the HB-chip was tested on patients with metastatic prostate cancer, CTCs were detected in 93% (14 of 15) of patients with counts ranging from 12 to > 3000 CTCs/mL with CTC clusters of 4–12 cells also identified, demonstrating the low sheer flow properties of the chip and the advantage of having a flat antibody contact surface. As no CTC clusters were observed in their first-generation CTC-chip, they hypothesised that the closely spaced microposts of the CTC-chip may have prevented the passage of CTC clusters [20]. 

The geometrically enhanced differential immunocapture (GEDI) chip uses geometrically enhanced microposts in an alternative way to disrupt laminar blood flow to provide greater contact opportunities between CTCs and immunocoated walls [21]. The chip contains 5000 octagonal shaped microposts coated with anti-PSMA (prostate-specific membrane antigen) antibodies, designed to capture CTCs from castrate-resistant prostate cancer (CRPC) patients, and combines this positive enrichment approach with hydrodynamic chromatography to increase CTC capture and reduce nonspecific leukocyte binding. They tested prostate cancer cell lines spiked into healthy blood and found 85 ± 5% capture efficiency and 68 ± 6% purity. They used the GEDI chip to successfully isolate CTCs from patients with CRPC and a direct comparison with the CellSearch showed a 2 to 400-fold increase in the number of CTCs captured using the GEDI chip compared to the CellSearch [82]. They then went on to develop a chip functionalised with HER2 antibodies to expand the clinical utility of the chip which when tested on metastatic breast and gastric cancer patients identified CTCs in 100% (9/9) of the samples [83].

The TAXYNERGY trial (NCT01718353) (Table 2) was a phase II study to evaluate the clinical benefit of an early taxane switch from docetaxel to cabazitaxel or vice versa in metastatic CRPC patients, which used CTCs isolated using the GEDI chip as a biomarker for treatment response [74]. When analysing post-treatment CTCs for their percentage of androgen receptor nuclear localisation (%ARNL), they saw a taxane-induced decrease in %ARNL. This confirmed the association between taxane drug-target engagement and clinical response and therefore suggested that %ARNL may prove to be an important early biomarker for patients treated with taxane therapy.

The HTMSU (microchip-based high-throughput micro sampling unit) chip is a more simplistic design chip with 51 high-aspect microchannels functionalised with anti-EpCAM (or anti-PSMA) antibodies for CTC capture [22]. The device is high throughput with 1 mL of whole blood processed in 2.7 min and a high capture efficiency of >97% from MCF-7 cells spiked into healthy blood is reported, with trypsin used to release unlabelled captured CTCs from the antibody coated surface. The novelty of this device lies in the enumeration where on-chip single-cell conductometric counting is used to detect the unique properties of single CTCs passed through the detection electrodes. This enumeration platform appears extremely advantageous since it negates the need for cell staining and manual microscopic counting. 

The Nanovelcro chip is one of four generations of its kind developed at the University of California, Los Angeles (UCLA), each generation developed with different clinical utilities in mind [23]. The principle behind this technology is its analogy to Velcro in terms of the ability for the anti-EpCAM coated silicon nanowire substrate (SiNS) to immobilise CTCs on the chip. The chip also contains a polydimethylsiloxane (PDMS) chaotic mixer on the roof of the chip to disrupt the laminar flow of blood thereby increasing contacts between CTCs and EpCAM antibodies for increased capture. The first-generation chip was created for simple CTC enumeration and the SiNS enables Velcro-like interactions with cell-surface proteins, however cells are not able to be recovered from the chip. Using spiked cancer cell lines, they achieved >85% capture efficiency [84]. When tested on blood samples from patients, the chip was able to detect CTCs in 100% of prostate patients (40/40), 75% of PDAC patients (54/72) and 94.7% of renal cell carcinoma (RCC) patients (72/76) [84,85,86].

The chip was used in a phase II study to evaluate caboxantinib (XL184) therapy in metastatic CRPC patients (NCT01834651) (Table 2). They used the Nanovelcro chip to look for very small nuclear circulating tumour cells (vsnCTCs) which they had previously identified in patients with visceral metastases and concluded that the Nanovelcto vsnCTC assay may be a predictive marker of response to caboxantinib therapy [75,87].

The second-generation chip (nanovelcro-LCM) was designed for single CTC-isolation. This chip uses the same anti-EpCAM coated nanosubstrates with an overlaid chaotic mixer but they designed a CTC capture polymer substrate that can be dissected using a laser capture microdissection (LCM) microscope to isolate single CTCs for downstream molecular analysis [88,89]. The group demonstrated successful whole genome sequencing (WGS) of four CTCs from a metastatic prostate cancer patient, with >95% coverage [90]. This chip however requires an initial RBC lysis step, which is controversial as it can result in the loss of CTCs. On top of this, the group reported two drawbacks of this chip to be the labour intensive process of the microdissection and generally low viability of recovered cells [23].

To address the issue of viability, the third-generation chip was designed used a thermoresponsive polymer on the SiNS [91]. CTC capture was still dependent on EpCAM antibodies, however the thermoresponsive polymer was effective at capturing and releasing CTCs at 37 °C and 4 °C, respectively, and resulted in high viability and molecular integrity of cells, allowing for downstream molecular analysis. At 4 °C, the polymer brushes undergo a conformational change and the anti-EpCAM antibodies are internalised, resulting in CTC release. When tested on NSCLC patients, the chip showed recovery rates of >70% and >90% viability of cells [92].

The fourth-generation chip discarded the thermoresponsive polymer and instead adopted a competitive binding approach to release captured CTCs [93]. The chip surface was coated with phenylboronic acid (PBA)-grafted poly(3,4-ethylene-dioxythiophene) (PEDOT)-based nanomaterial to which sorbitol competitively binds, resulting in the release of captured cells [94]. The group were able to perform RNA analysis on CTCs isolated from prostate cancer patients using this chip [93].

In an attempt to overcome the problem of low EpCAM sensitivity seen by the CellSearch, the IsoFlux (Fluxion) couples microfluidics with immunocapture, whereby the microfluidic approach results in increased capture efficiency due to increased contact between CTC-magnetic bead and magnetised surface. Whole blood is inputted into the machine where it is incubated with antibody coated magnetic beads, with up to four samples able to be processed in parallel. The blood is then flowed through a chip and CTCs are captured in the ‘isolation zone’ (an expanded cavity) on the upper surface of the chip which is exposed to an externally applied magnetic field [95]. Samples take 45 min to process and the upper surface of the chip containing the enriched CTCs is easily removed following enrichment, negating any cell loss that can occur during CTC recovery. The enriched CTCs are retained in a 3 μL droplet, which is ideal for downstream molecular analysis that often requires low sample volume for processing [4].

The company offers multiple isolation kits including EpCAM, EpCAM/EGFR and a mesenchymal isolation kit containing EpCAM/EGFR/Vimentin/N-cadherin antibodies to allow for sampling of the full heterogeneity of CTCs. Manual staining of CTCs is required post processing but the associated IsoFlux Cytation Imager allows for sample scanning in under 10 min and presents a panel of potential CTCs to the user for identification, as seen with the CellSearch CellTracks Analyser. The Isoflux is a robust platform and has been validated using thousands of samples across a wide range of cancers including colorectal, breast, prostate, ovarian, kidney, liver and bladder and has been shown to be able to recover CTCs from early-stage as well as late-stage disease [96,97,98,99,100,101,102].

There is evidence to suggest that the IsoFlux may be superior to the CellSearch including the application of the mesenchymal antibody kit and the microfluidic approach to increase the sensitivity of the device and to reduce the leukocyte background in the recovered sample. A direct comparison was carried out between the Isoflux and CellSearch using PC3 cells spiked into healthy blood with recovery rates of 90% and 40% respectively [4]. When comparing the two methods in matched prostate cancer samples, positive CTC counts (>4 CTCs) were observed in 95% (21 of 22) of samples processed using the IsoFlux compared to only 36% (8 of 22) samples processed with the CellSearch, suggesting greater sensitivity for CTC detection of the IsoFlux system.

One group combined IsoFlux CTC enrichment with droplet digital polymerase chain reaction (ddPCR) to analyse total androgen receptor (AR) and androgen receptor splice variant V7 (AR-V7) in prostate cancer patients [103]. In prostate cancer, genetic changes in the AR result in resistance to androgen deprivation therapy (ADT), which is the first line therapy for patients with metastatic disease [104]. The splice variant AR-V7 results in a truncated ligand binding domain, rendering the receptor constitutively active, independent of testosterone levels. Whilst AR-V7 confers resistance to ADT, it has also been identified as a biomarker for resistance in metastatic CRPC, therefore the ability to monitor levels of AR-V7 using a simple liquid biopsy could provide valuable information to clinicians [100]. The group initially performed experiments with cell lines spiked into healthy volunteer blood to determine the sensitivity of their ddPCR assay and concluded that conservatively, five cells in 4000 lymphocytes were required from a typical IsoFlux output to reliably detect AR and AR-V7 expression. They processed samples from 16 CRPC patients with the ddPCR assay following IsoFlux enrichment and detected AR-V7 in 50% (8 of 16) samples. AR-V7 detection rates were marginally higher than other groups who report detection rates ranging from 19% to 46% using other quantitative PCR (qPCR) AR-V7 assays which therefore may suggest a superiority of CTC isolation using the IsoFlux and/or a greater sensitivity of the ddPCR assay used [105,106,107].

Microfluidic immuocapture methods generally provide higher capture efficiencies than non-microfluidic devices, however they have the disadvantage of slower processing time. Irrespective of the potential for greater capture efficiencies, all of these immuno-magnetic microfluidic devices, apart from the IsoFlux, pose the same limitations as immunomagnetic capture methods in that the CTCs collected are biased towards epithelial-like cells. As a result, these devices have limited clinical applicability and are unlikely to be the next device to gain FDA approval. 

## 5. Capture Enhancement by Nanomaterials

Several groups have designed chips with integrated nanomaterials in an attempt to increase capture efficiency. The gold nanoparticle herringbone CTC chip (NP-^HB^CTC-Chip), graphene oxide (GO) chip and silica nanoparticle (SiNP) platform all combine nanoparticle addition with EpCAM antibodies for CTC capture. The NP-^HB^CTC-Chip is a herringbone chip coated in gold nanoparticles (AuNPs), which allows for the release of CTCs using an AuNP–thiol exchange reaction by the addition of glutathione (GSH) and provides greater capture efficiency and higher purity recoveries compared to the HB-chip [24]. The AuNP surface can be functionalised with EpCAM, HER2, EGFR antibodies or a cocktail of the three to enhance capture efficiency. The chip has been tested on patients with metastatic breast cancer however it is extremely low throughput, processing 3 mL whole blood in approximately 4 h, meaning the chip is not useful for high throughput clinical need [24].

The group used a patient-derived breast (Brx) CTC line to analyse gene expression profiles of CTCs before and after GSH release from the chip. Each blood sample spiked with Brx CTCs was split into control cells (for which RNA was extracted from cells on-chip following capture using the NP-^HB^-CTC-Chip) and released cells (for which RNA was extracted following ligand-exchange release of cells from the chip) [24]. Using reverse transcription qPCR (RT-qPCR), they looked at expression of EpCAM, Cdh3, HER2, MET and EGFR and showed identical threshold cycle (Ct) values for both groups of cells demonstrating that CTC viability and molecular signatures are maintained following the chemical GSH mediated release. Next-generation RNA sequencing also demonstrated negligible change in expression levels when looking at breast cancer-specific genes between the two groups of cells and they were also able to identify signatures related to progression, metastasis and EMT. Brx CTC lines are highly heterogeneous and more sensitive than an immortalised cell line, however it is important to remember that cultured patient-derived CTC lines will be more robust than CTCs in the circulation so it would be important to validate these results further with patient derived non cultured CTCs.

The GO chip is composed of flower-shaped gold patterns over which functionalised GO nanosheets are placed [25]. GO has a high surface area which when decorated with EpCAM antibodies results in a large surface area for CTC capture without the need for complex micropost geometry.

The group found average capture rates of 85% for cell line spiking experiments, spiking between 3 and 100 EpCAM^high^ MCF7 cells [25]. Similar spiking experiments performed with EpCAM^low^ PC3 cells resulted in capture rates greater than 65%, demonstrating good sensitivity of the chip. They also isolated CTCs from a small number of metastatic breast and prostate, and early-stage lung cancer patients. They successfully extracted RNA from CTCs isolated from breast cancer patients and were able to show HER2 gene amplification in 4/6 HER2 positive patients, demonstrating that the chip is able to isolate CTCs with sufficient quality RNA for subsequent downstream molecular analysis. 

As with many immunocapture methods, they initially faced difficulties with CTC release following capture but went on to develop a thermoresponsive chip to allow the simple release of viable cells through conformational changes in the polymer observed at 4 °C [26]. This modification results in higher capture and release efficiencies using cell lines of varying EpCAM expression levels (90% capture and 93% release), with the viability of released cells indicated as 91.68% using a live-dead assay. The chip has been tested on a small number of metastatic breast and prostate cancer patients with 67–80% capture efficiencies. The major benefit of this chip is the ease with which highly viable cells can be released for further downstream analysis.

The SiNP chip works on the same basis as the GO chip whereby anti-EpCAM-coated 10 μm silicananoparticles are used to coat the base of the chip, creating a three-dimensional capture platform. This provides a high surface area which ultimately allows for enhanced topographic interactions between the nanostructured substrate and CTCs, resulting in increased cell capture [27]. They achieved capture rates as high as 84–91% when spiking MCF7 cells into healthy blood and demonstrated this approach improves capture yields by >40% compared to flat Si substrate platforms.

The nanotube-CTC-chip utilises a novel approach whereby the 76-element array is composed of carbon nanotube (CNT) surfaces onto which CTCs preferentially adhere to and as a result, the use of EpCAM antibodies is not required. An initial RBClysis step is first required and following this, 10 μL droplets of pelleted nucleated cells are added to the chip. The cancer cells form strong focal adhesions to the nanotube surface resulting in a 5-log reduction of contaminating leukocytes, however the initial RBC lysis step is controversial as previously discussed. The group assessed different time points observing the percentage of adherence of CTCs and suggested that 48 h was optimal to give 90% cellular adherence [28]. This is too long if any downstream analysis of captured CTCs is required, however the planar enrichment surface makes microscopy and imaging post enrichment easy if only CTC enumeration is required. Nevertheless, this device is both label independent and size independent so is an extremely novel method of CTC capture even if some further optimisation is required.

## 6. Membrane Filtration

Microfilter membrane microfluidics are a more simplistic group of size-based CTC separation techniques. Such devices comprise simply a filter with pores of defined sizes and shapes through which blood is passed either using a pressure regulator or by centrifugation. Such devices are generally quick and easy to use which are both key characteristics when considering clinical applicability.

The flexible micro spring array (FMSA) contains round shaped pores of 8 μm diameter and flexible micro spring structures etched into a parylene filter. This is connected to a pressure regulation system which is used to gently pass the blood through the filter, maximising the viability of the recovered CTCs, with a reverse pressure applied to recover cells from the filter [29]. The device has been used to recover CTCs from breast, colorectal and NSCLC patients and a variety of different cell lines spiked into healthy blood gave 90% capture efficiency with 80% of cells remaining viable [29].

The FMSA was used in a trial to assess CTC isolation during resection of colorectal cancer (CRC) lung and liver metastases (NCT01722903) (Table 2). They showed that CTCs are enriched in the blood during CRC liver and/or lung metastasectomy and therefore perioperative liquid biopsy sampling creates an opportunity for increased CTC capture, providing the potential for greater success with downstream analysis to help identify personalised therapies [76].

The ScreenCell offers filters containing different sized pores of 7.5 μm or 6.5 μm randomly distributed throughout the filter for the isolation of fixed or live cells, respectively. The device requires an initial RBC lysis stage, however they claim near 100% recovery rates of cells and have a very quick processing time of 50 s for 1 mL blood [30]. The company offers three different devices depending on the downstream requirements which include the ScreenCell Cyto (fixed cells for cytological studies), the ScreenCell CC (live cells for cell culture) and the ScreenCell MB (for molecular biology requirements). Following filtration, the filters are easily released from the device by pushing a rod located at the bottom part of the device to release the CTCs on filter into any container desired by the user for specific downstream requirements. The ScreenCell MB device allows for the addition of lysis buffer to the capsule filter following filtration of blood to allow for DNA or RNA extraction directly on the filter [30].

The ScreenCell device has been used in the EXPEVIVO-CTC trial (NCT03797053) for the ex vivo expansion of CTCs from melanoma patients as a model for cancer predictive pharmacology and in the ESO-CTC trial (NCT02610764) to establish the relevance of CTCs in resectable oesophageal adenocarcinoma patients (Table 2), however no results have been published to date.

The filters are cheap and easy to produce but unevenly distributed or fused pores can drastically reduce capture efficiency. As with all filters, the ScreenCell is prone to clogging of the filter and blood must be first diluted prior to filtration. Spiking experiments were carried out with 5 and 2 NCI-H2030 cells to test for the sensitivity of the device, with an average recovery of 91.2% and 74% for five and two cells, respectively, demonstrating high sensitivity [30]. Another group isolated CTCs from 10 patients with primary lung cancer and CTCs were detected in 80% of patients prior to surgical resection [108]. The device has also been successfully used to isolate CTCs from melanoma and CRC patients and also from rare cancers including hemangiopericytoma [109].

The ISET (Isolation by Size of Tumour Cells) (Rarecells Diagnostics, Paris, France) consists of a design of 8 μm cylindrical pores and allows for 12 samples to be filtered in parallel. In contrast to other membrane microfilters, the ISET has a relatively slow processing time whereby 10 mL blood is processed within 4 h and blood must be diluted 1:10 to prevent membrane clogging. Once loaded, the sample is filtered by applying a vacuum resulting in gentle aspiration of the sample [31]. As little as 1 mL of blood can be processed, which may be advantageous as blood draws can often be difficult in cancer patients and they also claim high sensitivity of the device with the ability to detect as little as one single tumour cell spiked into 1 mL blood [110]. They have also developed the associated CTC-biopsy system, a semi-automated CTC detection system to allow for easy enumeration following filtration.

One group carried out a direct comparison between the ISET and CellSearch in 60 patients with metastatic breast, prostate and lung cancers, with concordant results obtained in only 55% (11 of 20) of breast, 60% (12 of 20) of prostate and 20% (4 of 20) of lung cancer patients [31]. The CellSearch outperformed the ISET in breast cancer patients whilst the ISET outperformed the CellSearch in prostate and lung cancer patients and in total, 30% of patients (18 of 60) were found to have negative CTC counts with the CellSearch, whilst only 5% (3 of 60) of patients were negative with the ISET. The variability in concordance seen here clearly shows the limitations of EpCAM-based enrichment methods and also the underestimation of CTCs processed using the CellSearch. Another group compared CTCs from RCC patients using the CellSearch and ISET [111]. The CellSearch has only a 10–20% detection rate in RCC patients and the ISET showed detection rate of 36.1%. Other studies have shown the ISET is better than the CellSearch at detecting CTCs in patients with NSCLC, pancreatic, oesophageal and metastatic prostate cancer. 

The ISET features in several clinical trials (Table 2), most notably the IMMUNO-PREDICT trial (NCT02827344) and the STALKLUNG01 trial (NCT02372448). The IMMUNO-PREDICT trial analyses PDL-1 expression on CTCs isolated from NSCLC patients, the detection of which would allow the stratification of patients for PDL-1 inhibitor therapy, negating the requirement for invasive biopsies. The STALKLUNG01 trial was designed to detect ALK gene rearrangements in CTCs, allowing patients with inoperable NSCLC to benefit from crizotinib treatment in instances when tumour biopsy is not feasible. Since approximately 30% of tumour biopsies contain insufficient material for ALK molecular characterisation, they concluded that CTC analysis could effectively be used in parallel with tumour biopsy analysis to allow a more complete identification of patients who would benefit from ALK inhibitor therapy [77]. Aside from lung cancer, the ISET also features in clinical trials for colon and rectal cancers (NCT02554448, NCT02979470), bronchial cancer (NCT03328559), malignant pleural mesothelioma (NCT01776385) and prostate cancer (NCT04702633).

The separable bilayer (SB) microfilter contains two layers of parylene-C filters, which is reported to preserve cell viability, with pores arranged hexagonally and aligned top and bottom. The top and bottom layers contain pores of 8 μm and 40 μm, respectively, with the idea that CTCs become trapped between the two layers causing minimal mechanical stress to the cells [33]. Following enrichment, the parylene-C membranes can be easily separated to recover captured cells. Unfortunately, the filter is only able to process 1 mL of blood at a time due to device clogging and due to the rarity of CTCs in the blood multiple filters from the same patient would likely be required to generate any meaningful results. However, the device was initially tested on various cancer cell lines spiked into healthy blood and gave a capture of efficiency of 78–83% with 71–74% of cells remaining viable. The filter was then further tested on blood samples from metastatic CRC patients, however they concluded the current filtration area would need expanding in order to facilitate processing of larger clinical samples of 7.5 mL [33].

The fluid assisted separation technology (FAST) lab-on-a-disc platform (Clinomics, Ulsan, Korea) has been designed to try and combat the common problem of filter clogging by introducing an aqueous phase held in a chamber below the filter [112]. The filter contains 8 μm pores and the aqueous phase immediately below this causes the blood to uniformly diffuse through the membrane. A centrifugation step acts as the final step to isolate and retain CTCs on the filter. The filtration phase is extremely quick with 3 mL blood filtrated in >1 min. The device has been tested on breast, lung and gastric cancer cell lines with a broad range of EpCAM expressions, giving recovery rates of 96.2% ± 2.6% with a 2.5 log depletion of leukocytes observed. They then went on to test blood from breast, stomach and lung cancer patients with clinical detection rates of 83.3% (15 of 18), 82.9% (63 of 76) and 68.6% (24/35), respectively [34].

Although filtration-based techniques are simple to use, they are generally fairly low throughput and are only able to process small volumes of diluted blood at a time and are prone to membrane clogging. They also generally result in high leukocyte contamination and therefore low levels of purity. With no specific way to efficiently remove CTCs from the membrane, a simple washing step could result in huge loss to CTCs and may not be a viable step for cell recovery. Such filtration technologies offer the possibility for cell staining on the filtration membrane or filters can be placed directly into a cell culture dish for cell culture or into a recovery tube for pooled DNA/RNA extraction, if a high leukocyte background is acceptable. However, if downstream single cell analysis is required then a different enrichment technique should be used.

## 7. Size-Based Microfluidics

Size-based microfluidic devices are likely to pave the way for the next generation of CTC enrichment technologies due to their separation occurring independently of cell surface markers, which in theory allows the capture of the full heterogeneous population of CTCs from any type of cancer. These devices are based on the knowledge that CTCs (~8–30 μm) are generally larger than leukocytes (~12–15 µm) and are less deformable than other blood components. Since there is some degree of crossover between CTC and leukocyte size, it is crucial that size-based microfluidic devices are optimised in order to maximise CTC recovery rates whilst minimising inevitable leukocyte contamination rates.

The Parsortix (Angle) device has been developed with a chip containing a stepped/gradiated separation structure that gradually decreases with size, with final gap sizes ranging from 4.5 μm to 10 μm. The most common GEN3D6.5 Cell Separation Cassette has a “critical gap” size of 6.5 µm which acts to capture larger CTCs and allows other smaller, more deformable blood components to pass through [113]. The device is fairly slow at processing, taking ~4 h for 7.5 mL blood and is only semi-automated, requiring significant user input. Following cell capture, a reverse pressure is then applied to harvest the CTCs. The device is able to capture CTC clusters and there is the option for on-chip staining, however on chip imaging is difficult. Using the 6.5 µm gap size cassette, an average capture rate of 62.4% was achieved across breast- and NSCLC-derived cell lines, however purity decreases as the cassette “critical gap” size decreases [35]. The system is well suited to enrich CTCs from clinical samples (although harvested cells are not pure CTCs), taking advantage of the different physical properties (i.e., size and deformability) of the target rare cells compared to other blood components such as RBCs and WBCs.

The Parsortix is used in several clinical trials (Table 2) including those for NSCLC (NCT03771404), breast (NCT03427450), prostate (NCT04021394) and ovarian cancers (NCT02781272, NCT02785731), however no results have been published to date.

The microcavity array (MCA) contains a polymethyl methacrylate (PMMA) filtration cartridge with the filter composed of nickel and gold. In total, 10,000 cavities are arranged in a 100 × 100 array. Cavities have a diameter of 8–11 μm and are spaced 60 μm apart, with a negative pressure applied to the device resulting in CTC capture [36]. Staining can be done on-chip and each device can process four independent samples in parallel. The device has been shown to efficiently capture CTCs from NSCLC patients. A direct comparison with the CellSearch identified 77% (17 of 22) of patients as CTC positive using the MCA and 32% (7 of 22) using the CellSearch. From matched patients, the MCA detected a median of 13 CTCs (range 0–291 cells/7.5 mL) and the CellSearch detected a median of 0 CTCs (range 0–37 cells/7.5 mL) [114].

An alternative group of devices exploit lift forces imposed on CTCs as they flow through the chip due to their differential biomechanical properties compared to other blood components. The ClearCell FX1 platform (Biolidics, Singapore) is a spiral microfluidic device which uses Dean Flow Fractionation (DFF) principles (dean forces and lift forces) to focus and retain larger CTCs at the inner side of the spiral channel whilst the remaining blood components flow through the chip via the outer side of the channel [115]. This device is relatively quick and can process 7.5 mL blood in 1 h, however an initial RBC lysis step is required. The technology is optimised to capture CTCs of approximately 14 μm in diameter, however they also give the option to capture smaller CTCs by changing the flow rates if a greater leukocyte contamination is acceptable [37]. The device releases label free, viable cells, available for downstream processing such as next-generation sequencing (NGS) or proteomics [116,117]. The channel dimensions allow for high throughput processing with no channel clogging as is seen with many other devices.

One group used the ClearCell device to detect CTCs in 77 blood samples (56 cancer patients, 21 healthy volunteers) with 80.4% sensitivity and 85.7% specificity, with purity on average 20,000 leukocytes per 7.5 mL blood [37]. Others validated the platform with MCF-7 and H1975 cell lines spiked into healthy volunteer blood. They spiked varying numbers of cells ranging from 50 to 2000 cells per 7.5 mL blood and obtained recovery rates of 64.5% ± 12.34% and 69.9% ± 9.74% for MCF-7 and H1975 cells, respectively, however this is not necessarily an indication of expected recoveries with clinical samples due to the rare nature of CTCs in the blood. When they tested the ClearCell FX1 on patients with both primary and metastatic breast cancer, they detected CTCs in 81.3% (26 of 32) of patients with primary and 73.7% (56 of 76) of patients with metastatic breast cancer, indicating good clinical detection rates [38]. However, this group viewed a positive CTC count as ≥2 CTCs per 7.5 mL blood, which is contrasting to the widely accepted positive count of ≥5 CTCs per 7.5 mL established as the CellSearch threshold. Another group compared CTC recovery using the ClearCell FX1 and CellSearch and saw similar recovery rates from high EpCAM expressing cell lines of 67% ± 11% and 74% ± 10%, respectively, but saw much greater differences in recovery of low EpCAM expressing cell lines with rates of 62% ± 8% and 32% ± 9% for the ClearCell FX1 and CellSearch devices respectively [38].

The ClearCell FX system features in clinical trials for lung NCT02370303) and head and neck cancer patients (NCT04696744) (Table 2), however results are yet to be published.

The VTX-1 technology (Vortex) utilises similar principles. The chip consists of 16 channels containing 12 rectangular reservoirs per channel with filters located at the channel inlets to prevent clogging [39]. As the blood flows through the channels, micro-scale vortices are generated within the reservoirs which expose the larger CTCs to shear gradient lift forces which trap the CTCs within the microvortices in the reservoir. Following a washing step, the buffer flow rate into the chip is simply lowered which dissipates the vortices and releases CTCs for collection [118]. This system is fully automated and blood is processed within 1–2 h with no initial centrifugation step required. The associated BioView platform allows for automated cell imaging and, much like the CellTracks Analyser for the CellSearch, presents a panel of potential CTCs to the user for review and enumeration. The BioView also gives the additional option for bleaching of CTCs following identification to allow for further analysis such as fluorescence in situ hybridisation (FISH) [40].

The device has the option to run in two modes: “high recovery mode” or high purity mode”. In the high recovery mode, the sample is processed up to three times to increase CTC capture but results in a lower purity sample. The high purity mode processes the sample just once resulting in a higher purity sample but with a lower efficiency of CTC capture compared to multiple rounds of processing. The two modes were tested using 50 MCF-7 cells spiked into 4 mL healthy blood. The high recovery mode gave 71.6% recovery with 350 contaminating leukocytes per ml blood and the high purity mode gave 53.8% recovery with 101 contaminating leukocytes per ml blood processed [40]. The device has been validated, for research purposes, for use in metastatic breast, colon, lung and prostate cancers and they demonstrated that recovered cells were highly viable and ideal for cell culture experiments, live cell assays and RNA analysis to name just a few. CTCs can be collected in whatever container the user requires for downstream use, meaning no transfer is required which could result in the loss of CTCs. 

One group performed targeted analysis of *KRAS*, *BRAF* and *PIK3CA* mutations in CTCs from metastatic CRC patients isolated using the VTX-1 [119]. Mutational status of *KRAS* and *NRAS* are routinely assessed in CRC patients to determine whether EGFR inhibitors such as cetuximab may provide clinical benefit following surgical resection [120]. The ability to assess this in CTCs could provide important insight into the benefits of such treatment, since *RAS* status may be discordant between primary tumour and subsequent metastases and also when considering the difficulty of obtaining a biopsy from certain metastatic sites. They performed PCR-based sanger sequencing, which was shown to successfully detect mutations in samples with a purity of ≥7.5%. They were able to isolate CTCs using the VTX-1 with a mean purity of 14.5% and detected at least one mutation in 78% of the samples with 77.8% of the samples showing concordance with tumour biopsies [119].

The parallel multi-orifice flow fractionation device (p-MOFF) chip is composed of four single MOFF channels in parallel, which each contain a series of contraction/expansion microchannels whereby inertial forces concentrate cells along the walls according to size. Since WBCs are smaller than CTCs and are influenced less by inertial lift force from the series of contraction/expansion channels, they become focused towards the outside edges of the channels, with CTCs focused at the centre of the channels [41]. The downfall of this is that smaller CTCs of a similar size to leukocytes will be discarded. The device is high throughput and able to process 7.5 mL blood in 30 min, however RBC lysis and Ficoll density centrifugation steps are firstly required. The outlet flow rate has been engineered to be 40% of the total inflow rate to enable greater CTC recovery. When spiking MCF-7 and MDA-MB-231 cells into healthy blood, recoveries of 93.75% and 91.6% were observed, respectively. Blood samples have also been analysed from breast cancer patients using the device, with CTCs detected in 90.5% of patients and numbers ranging from 1–21 CTCs [41]. 

As previously mentioned, size-based microfluidics methods are advantageous over many well designed immunocapture methods due to their label free capture design. Such an approach allows for the capture of both mesenchymal and epithelial CTCs, allowing for the sampling of the heterogeneous population of CTCs, as well as broadening the clinical utility to enable the capture of CTCs from all cancer types, not limited to just epithelial cancers. They are also generally cheaper to produce due to the lack of expensive labels, however they are often fairly low throughput with slow processing times coupled with the limitation of most devices to be able only to process one sample at a time. There is a fine line when deciding the size of CTCs to be captured as some smaller CTCs often overlap in size with leukocytes, especially in cancers such as breast with typically smaller CTCs. Microfluidic chips can be designed with smaller channel widths to overcome this problem, only if a high level of leukocyte contamination is acceptable. In such an instance, the user could process enriched samples, regardless of the leukocyte background, on the DEPArray (Menarini Silicon Biosystems), discussed later, to recover either single CTCs or pools of CTCs with 100% purity.

## 8. Density Based

Density-based separation methods make up the more simplistic class of CTC enrichment technologies. They were one of the first types of CTC separation techniques developed and as technology has evolved, these methods are generally not viewed as the most efficient of the CTC separation techniques. The OncoQuick (GrenierBioOne) combines density centrifugation with filtration in a centrifugation tube containing a micro-filter placed above liquid density separation media [121]. Blood is layered on top of the gradient and centrifugation results in CTC capture on the filter. Up to 25 mL blood can be processed per tube, however a wide variety recovery rates have been reported between 25 and 80% [43,122]. In a direct comparison with the CellSearch, processing blood from 61 patients with multiple different cancer types, the OncoQuick detected at least 1 CTC in only 23% (14 of 61) of patients, compared to 54% (33 of 61) of patients analysed using the CellSearch [123].

The AccuCyte (RareCyte; Seattle, WA, United States) system utilises a unique separation tube which contains a lozenge-shaped float. Following centrifugation, the hollow cylindrical float rests at the blood cell-plasma interface to allow collection of the buffy coat within the float [124]. The plasma is then manually aspirated from the sample before a collection device (EpiCollector) is placed on top of the separation tube. A transfer tube containing high density retrieval fluid is placed into the EpiCollector and another round of centrifugation displaces the buffy coat into the transfer tube. The buffy coat is retrieved from the transfer tube and evenly distributed onto a microscopic slide using the CyteSpreader, a manual spreading device, before on-slide automated immunohistochemistry (IHC) staining is performed using antibodies against EpCAM, EGFR, CD45 and Hoechst 33342 or DAPI. It does not require any washing steps which helps to minimise CTC loss. The microscopic slide is then scanned using the CyteFinder system, an automated scanning digital microscope which couples with the image analysis software CyteMapper to present images of potential CTC candidates to the reviewer much like the CellSearch associated CellTracks analyser [44]

Integrated within the CyteFinder system is the CytePicker which allows the mechanical selection of individual CTCs for recovery and further downstream processing. The total processing time from sample to cell picking including IHC staining is 7 h with only ~1 h hands on time, which is similar to the processing time for the CellSearch-DEPArray workflow. The AccuCyte system also allows for the processing of multiple samples in parallel, as with the CellSearch. 

One group tested recovery rates of the system by spiking LNCaP and PC3 (prostate), A549 (lung) and MCF7 and SKBR3 (breast) cell lines into 7.5 mL healthy whole blood [44]. Spiking ~100 cells, they observed an average recovery rate of 90.5% with minimal deviation of average recovery rates between EpCAM^high^ (LNCaP, MCF7) and EpCAM^low^ (PC3, A549) expressing cell lines. Low number spiking experiments were also performed spiking 1–6 PC3 cells into 7.5 mL blood for which they obtained an average recovery rate of 81%. Additionally, they showed the device was able to detect just a single cell in 7.5 mL blood demonstrating high sensitivity of the device.

They successfully performed Whole Genome Amplification (WGA) on eight individual and one pool of five SKBR3 cells using the *Ampli1* WGA kit (Silicon BioSystems) and clearly detected *TP53* R175H mutations in all samples by both PCR followed by Sanger sequencing and whole exome sequencing (WES).

They also performed a direct comparison with the CellSearch using clinical samples from 10 advanced breast, prostate or CRC patients. In three of the paired samples, the AccuCyte recovered significantly more CTCs than the CellSearch. In the remaining seven paired samples, both methods recovered similar numbers of CTCs, with very low numbers (≤3 CTCs) detected in four of these seven samples using both methods). The AccuCyte is a highly developed system with the associated CyteFinder and CytePicker for CTC enumeration and single cell isolation, respectively. They demonstrated that recovered cells remain viable for single cell WGA and further downstream molecular analysis. It is clear the AccuCyte system is a worthy competitor for the CellSearch, with the advantage that it is a label free separation device, enabling the isolation of both epithelial and mesenchymal CTCs.

Density-based separation techniques have the advantage of quick processing time due to just a simple centrifugation step required, however they often result in low purity enrichments, and the requirement for post processing staining and microscopic visualisation for manual enumeration adds considerable time to the process [125].

## 9. Dielectrophoresis

Other enrichment methods use dielectrophoretic field forces to move CTCs independently of other blood cells. This is advantageous as it is label free so separates cells independently of EpCAM expression and is highly specific, however expensive. The ApoStream technology (ApoCell) couples dielectrophoresis (DEP) with field-flow assist for the isolation of CTCs [126]. The device requires an initial Ficoll separation step to isolate PBMCs which are then processed using the device. The cells are passed over a DEP-field in a laminar stream and their alternative current frequency pulls CTCs to the floor of the chamber out of the stream as they pass over the electrode, whilst other blood components continue to flow through. The iCys laser scanning cytometer is used for CTC enumeration following enrichment [45]. The DEP separation is able to separate CTCs from the buffy coat from 7.5 mL blood in approximately 60 min, giving good recovery rates with high purity and high viability of recovered cells [46].

The device was validated using A549, MDA-MB-231 and ASPS-1 cell lines with recoveries ranging between 55% and 68% depending on the cell line, with MDA-MB-231 cells recovered with 97% viability [127]. Another group tested the device with SKOV3 and MDA-MB-231 cells (high and low EpCAM expression, respectively) with recoveries of 75.4% ± 3.1% and 71.2 ± 1.6%. They also demonstrated high sensitivity of the technology and were able to recover two cancer cells from as few as four spiked into healthy blood which is extremely important when considering the rare nature of CTCs in the blood [46].

The ApoStream features in two phase I clinical trials to isolate CTCs from patients with advanced solid tumours (NCT02466802) and early-stage pancreatic cancer (NCT02349867) (Table 2), however results including CTC characterisation are yet to be published.

## 10. In Vivo Enrichment

Diagnostic leukapheresis (DLA) involves the screening of litres of whole blood and can be used as a pre-enrichment step to overcome the limited numbers of CTCs often found in a small blood draw. DLA involves the continuous flow centrifugation of blood, which is commonly used to isolate PBMCs with a density of 1.055–1.08 g/mL. Since epithelial cells have a similar density to PBMCs, CTCs will also be enriched in the DLA product [128].

A direct comparison was carried out in breast cancer patients using the CellSearch to enrich CTCs from matched DLA samples and standard 7.5 mL peripheral blood draws [47]. Approximately 40 mL of DLA product was collected from patients (median blood volume processed 2.77 L) and a small aliquot (~5%) of this was processed using the CellSearch, alongside 7.5 mL peripheral blood. In metastatic (M1) patients, CTCs were detected in 80% (12/15) of DLA samples compared to 71% (10 of 14) of peripheral blood samples. In non-metastatic (M0) patients, CTCs were detected in 55% (11 of 20) of DLA samples compared to 15% (3 of 20) of peripheral blood samples, suggesting DLA may provide greater benefit as a pre-enrichment method for patients with early-stage disease when CTCs are notoriously more difficult to detect. Considering that only ~5% of the DLA product was processed using the CellSearch, it was calculated that up to 43,156 (median 82.59; range 0–43,156) CTCs were collected from M1 and up to 1148 (median 12.35; range 0–1148) CTCs were collected from M0 patients, clearly demonstrating the ability for DLA to overcome the issue of the rarity of CTCs. Importantly, no adverse events were reported for patients during the DLA procedure. Subsequent enrichment of DLA product is not limited to use with the CellSearch platform, with other groups coupling DLA with the ISET and parsortix to successfully isolate CTCs [129,130,131]. 

The GILUPI CellCollector is a novel in vivo CTC enrichment device designed to overcome the limitations faced by in vitro devices in trying to isolate such rare cells from often small samples of blood. It is an anti-EpCAM coated wire which is inserted into the vein of patients via an intravenous cannula for 30 min to allow for the direct sampling of CTCs from the peripheral blood of cancer patients [48]. This allows for the screening of between 1 and 3 L of blood, which should yield higher numbers of CTCs. Validating in vitro experiments showed the purity of isolated cells to be >90% [132]. The device was initially validated in breast and NSCLC patients where they isolated CTCs from 91.6% (22 of 24) of patients from all tumour stages, including early-stage cancer patients where CTC detection is often challenging [48]. The device is naturally a more invasive procedure for the patient compared to a simple blood draw, however they reported that all patients tolerated the procedure with no adverse events.

Another group performed a direct comparison between the GILUPI CellCollector and the CellSearch in 80 CRC patients, however they saw no significant difference in the CTC detection frequencies between the two methods. They hypothesised that this was potentially due to an overestimation of the volume of blood sampled within the 30 min. They performed 2D and 3D in silico approximation experiments considering differences in anatomy, venous vasculature and blood flow rates of patients. They concluded a 30 min incubation period was more likely to sample a blood volume between 0.33 and 18 mL, as opposed to 1–3 L and therefore a much longer incubation period would be required to see significantly greater numbers of CTCs captured [133]. 

The GILUPI CellCollector features in clinical trials for early-stage breast (NCT03732339) and lung cancers (NCT02507778) and in a phase III trial to validate and evaluate the safety of the CellCollector in Chinese patients presenting with metastatic breast cancer (NCT03006055) (Table 2).

Such in vivo approaches are naturally more invasive for patients than a simple blood draw, however their ability to obtain increased numbers of CTCs could prove to be invaluable when considering the implications they could have for downstream processing. When considering the loss of CTCs that occurs during all stages of processing, including the initial enrichment steps, IHC staining and any further single cell isolation procedures, starting with greater numbers of CTCs will ensure that any loss that occurs during processing will not prove detrimental to downstream analysis requirements.

## 11. Combined Methods

The CTC-iChip is the third-generation chip developed by MGH (following the CTC-chip and HB-chip) and uses a combination of several approaches in the enrichment of CTCs [134]. Whole blood is initially incubated with magnetic beads functionalised with either EpCAM antibodies for a positive enrichment approach or CD45, CD16 and CD66b antibodies for a negative enrichment approach. The second ‘debulking step’ utilises deterministic lateral displacement to separate nucleated cells from RBCs and platelets. The cells are then passed through a microfluidic channel aligned in a near-single file line using inertial focusing, where they are passed over a magnetic field for the final magnetic enrichment approach. The idea of inertial focusing to align the cells while they are passed over the magnetic field provides increased sensitivity for the device and requires minimal magnetic force for separation, resulting in higher yielding and higher purity recoveries [135]. The device is high throughput, processing 8 mL blood per hour and high recovery rates were demonstrated, with the device also able to recover CTC clusters. They were able to recover viable, preserved cells and were able to perform transcriptomic analysis, drug screening and cell culture.

They spiked cell lines with a range of EpCAM expression into healthy volunteer whole blood and isolated CTCs using both positive and negative enrichment methods [49]. Using the positive enrichment approach, they demonstrated recoveries of 98.6% ± 4.3%, 89%.7 ± 4.5% and 77.8% ± 7.8% for SKBR3, PC3–9 and MDA-MB-231 cells, respectively. For the negative enrichment method, they spiked epithelial parental MCF10A cells and their mesenchymal derivatives MCF10A-LBX1 cells into healthy blood. They achieved CTC recoveries of 96.7% ± 1.9% and 97.0% ± 1.7%, respectively, therefore demonstrating good recoveries using both positive and negative enrichment methods. Purities of samples recovered from each selection method were 1500 WBCs/mL of whole blood (>3.5 log purification) for the positive enrichment approach and 3200 WBCs/mL of whole blood (2.5 log depletion) for the negative enrichment approach [49]. Further examination into this showed that the majority of WBCs remaining following positive selection were attached to magnetic beads, suggesting nonspecific binding, while the majority of WBCs remaining following negative selection were free cells, suggesting that these cells may exhibit low expression of the leukocyte depletion markers.

A direct comparison was also carried out between the CTC-iChip and CellSearch using blood samples from 19 prostate, 12 breast, 6 pancreas, 2 colorectal and 2 lung cancer patients. Both devices gave similar numbers from patients exhibiting a high CTC count (>30), however in patients expressing low CTC counts (<30 (86% of the patients)), the CTC-iChip isolated significantly more CTCs in 61.1% of the patients (22 of 36). This shows the sensitivity of the device to detect lower numbers of CTCs compared to the CellSearch, which is a critical factor when considering clinical applicability [49].

Most recently, the fourth generation MGH microfluidic chip has been published; the ultrahigh-throughput microfluidic ^LP^CTC-iChip which combines leukapheresis with high precision microfluidic negative enrichment to allow for the sorting of unlabelled, viable CTCs [50]. 

Leukepheresis concentrates on average 7 × 10^9^ PBMCs (including CTCs) from ∼5 L blood into a leukopak of ∼65 mL during an hour long procedure which is estimated to contain between 100 and 20,000 CTCs depending on the type and stage of disease [50,136]. The ^LP^CTC-iChip is able to process the entire 65 mL leukopak and allows for the depletion of between 50- and 100-fold more WBCs than other magnetic enrichment technologies. Following leukapheresis, leukocytes are labelled with a cocktail of biotinylated antibodies targeting CD45, CD16, CD3, CD45RA and CD66b before the sample is firstly flowed through an inertial separation chip. This depletes RBCs and platelets from the leukopak due to their smaller size [137]. After debulking, the resultant antibody-labelled WBCs are incubated with streptavidin-coated superparamagnetic beads before being processed using the magnetic ^LP^CTC-iChip.

In the chip, cells enter the stage-1 sorting channel via two asymmetric serpentine channels which align cells in a single file in the centre of the channel due to shear-induced lift and Dean flow-based drag forces. The chip contains external neodymium iron-boron magnets with modified polarity to direct the magnetic force towards the centre of the channel and internal soft iron-filled channels aligned either side of the sorting channels which act as magnetic microlenses, increasing the magnetic field gradient 35-fold. As the cells flow through the stage-1 sorting channel, the magnetic bead-coated WBCs are directed to the centre of the channel where they are funnelled into a waste port, whilst the unlabelled CTCs flow close to the walls of the channel where they are retained within the system and flow into the stage-2 sorting channel. Due to the high numbers of leukocytes in a typical leukapheresis product, the consistent funnelling of WBCs in a single cell stream through the centre of the channel ensures channel clogging does not occur and allows for high sensitivity, high throughput sorting. Cells then enter the stage-2 sorting channel (identical to the stage-1 channel) which increases the purity of the final output. Following leukapheresis, CTC enrichment takes 3 h, with the chip depleting approximately 3 × 10^9^ leukocytes per hour [50].

The group initially tested the chip by spiking 1000 ex vivo-cultured CTCs into leukapheresis mimic samples (representing approximately 1/3 of a normal clinical leukapheresis product). They recovered 89.2 ± 5.7% spiked CTCs and removed 99.96% WBCs (3.35 ± 0.17 log_10_ depletion) (*n* = 5). They then performed similar experiments spiking 5000 cells into healthy clinical (whole) leukapheresis samples (*n* = 3). They recovered 86.1 ± 0.6% of CTCs with an average purity of 0.3% (3.55 ± 0.26 log_10_ WBC depletion). Recovered CTCs retained their morphology and when cultured in vitro exhibited similar proliferative capacity to control cells. Using a ddPCR assay, they also demonstrated that enriched cells retained RNA of sufficient quality to allow for further downstream analysis. They finally assessed recovery when spiking low numbers of differing cell lines (ex vivo-cultured CTCs, MDA-MB-231 and LNCaP cells). For each of these lines, five cells were spiked into leukapheresis mimic samples (*n* = 5), with four or five cells successfully recovered each time, displaying the extremely high precision recovery ability of the chip.

One of the main drawbacks of working with CTCs is their rare nature within the blood. Since the processing of CTCs will almost always inevitably result in some cell loss, it can be difficult to gain meaningful results from a blood draw of only 7–10 mL. The clear advantage of the ^LP^CTC-iChip is its ability to process leukapheresis products which allow the sampling of 5 L of blood, which ultimately will allow for the recovery of sufficient numbers of CTCs to provide clinically meaningful results. It also may provide an avenue into CTC detection and analysis in early-stage cancer patients, who naturally have very low levels of CTCs [138,139]. Since a negative enrichment method is used, the chip can be used for all tumour types and the simple design of the chip means it is easy to produce and can be scaled up easily.

The authors have noted the limitations of the chip, acknowledging that CTC loss could occur during sample transfer between the two debulking and enrichment chips and plan to integrate both chips into one during the next-generation development. They also acknowledge that since the chip uses a negative depletion strategy, any CTC-WBC clusters will be lost. As a result, they plan to also incorporate a size-based CTC cluster chip to firstly isolate any CTC clusters prior to enrichment with the ^LP^CTC-iChip [140].

The On-chip Post processing Enabling Chip (OPENchip) device couples EpCAM-based microfluidic immunocapture with in situ molecular profiling that allows for simultaneous RNA and DNA analysis using image-based rolling circle amplification through the use of padlock probes designed to target biomarkers of interest [51]. This device that couples both CTC enrichment and on-chip downstream molecular analysis is unique to all other CTC enrichment devices which require independent molecular analysis following enrichment. The disadvantages to this technique are extremely low throughput at 1 mL per hour and only 50% recovery rate is reported due to intermittent washing steps. The device was validated using pancreatic, breast and colon cancer cell lines [51].

Overall, these combined methods are unique to other enrichment methods, with the ^LP^CTC-iChip demonstrating great promise for the enrichment of CTCs from early-stage as well as late-stage cancers. Since the CTC-iChip and OPENchip use immunomagnetic enrichment, they face the same problem of EpCAM bias within the recovered population as seen with other immunocapture methods. The CTC-iChip does present the possibility of a negative enrichment, however as previously discussed, negative enrichment methods often result in the inadvertent loss of CTCs and will also remove CTC-WBC clusters from the recovered population.

## 12. Secondary Isolation Technologies

With the aforementioned enrichment technologies, a degree of leukocyte background always remains, making the molecular analysis of CTCs challenging. Depending on downstream analysis application requirements, it may be beneficial to include a further isolation step to remove any contaminating WBCs, whose DNA/RNA may act as an obstacle for the precise molecular characterisation of CTCs. A secondary isolation step would provide users with either single CTCs to allow for the study of intrapatient heterogeneity, or pure CTC populations [141]. In these instances, primary enrichment methods should be coupled with single cell sorting technologies, some of which are already used in conjunction with primary enrichment technologies and have already been mentioned above. The most notable of which include manual micromanipulation, the ALS CellCelector and the DEPArray.

Manual micromanipulation involves the manual picking of stained CTCs using a microinjector controlled by a micromanipulator, with the process visualised using an inverted microscope [142]. Since it is a manual procedure, the process can be somewhat time consuming, however it allows for the dissociation of CTC clusters and CTC-WBC clusters into single cells to allow for subsequent single cell analysis of intra-cluster heterogeneity. Interestingly, it was shown that CTCs isolated from CTC clusters displayed greater survival and proliferative capacity compared to single CTCs [143].

The CellCelector technology (Automated Lab Solutions; Jena, Germany) is an automated micromanipulation platform and employs a high-precision glass micro-capillary fixed to a precision robotic arm [144]. Much like with the CellTracks Analyser, the sample is scanned and automated identification detects putative CTCs for individual or pure, pooled collection. The system is able to select individual cells from large numbers of contaminating cells and has been successfully used downstream of a wide range of primary enrichment methods [95,145,146,147,148].

The DEPArray (Menarini Silicon Biosystems) is a microchip-based digital sorter, which combines microfluidics with dielectrophoresis to allow for the isolation of single CTCs from a heterogeneous sample [149]. A volume of 12 µL of pre-enriched sample is loaded into a single-use microelectronic silicon chip, which integrates an array of >300,000 micro-electrodes. These micro-electrodes are used to generate up to 30,000 “DEP cages” in the Main Chamber of the chip, with each cage holding one individual cell. The Main Chamber has a volume of 9.26 µL, therefore the dead volume of 2.74 µL gives a final loading yield of 77% [150]. The DEPArray NxT, the latest development of the technology, allows the visualisation and acquisition of images using up to five fluorescent channels. This, coupled with bright field filter visualization, allows for the accurate selection of cells based on cell size, shape, circularity and fluorescence intensity.

Once the cells of interest have been selected and confirmed by the user, the DEP cages containing the selected cells are automatically moved by changing the electric field pattern step by step along calculated trajectories into the Parking Chamber [151]. Once cells have been successfully parked, the Main Chamber is washed and cells are subsequently moved to the Recovery Chamber from where they can be displaced as single cells or in pools of up to 507 cells [150].

The DEPArray has been developed by Menarini Silicon Biosystems as part of a standardised, reproducible workflow involving CellSearch primary enrichment, DEPArray single cell sorting and *Ampli1* WGA analysis for the accurate molecular characterisation of heterogeneous single CTCs [152]. Use of the workflow has been demonstrated in the analysis of copy number aberration (CNA) profiles of CTCs in small cell lung cancer cases to predict whether a patient will be chemosensitive or chemorefractory (relapsing within 3 months of initial treatment) [153]. Notably, the DEPArray technology can be used downstream of any primary enrichment technology and the opportunity to visualise and select cells using five fluorescent channels gives the technology tremendous versatility, providing options for isolation of both fixed and live CTCs.

## 13. Conclusions

At the time of writing, the CellSearch remains the only FDA approved technology to date. It has the ability to process multiple samples in parallel, coupled with in-device staining and automated imaging of cells that reduces both the hands-on time for the user and the overall processing time. However, as discussed, the CellSearch has a relatively low sensitivity to EpCAM, since cells expressing lower levels of EpCAM are not efficiently detected by the platform [3]. Microfluidic immunocapture methods that flow blood over EpCAM coated walls or microposts offer greater EpCAM sensitivities due to the increased contact between CTCs and EpCAM antibodies.

In recent years, mesenchymal CTCs have been shown to be more aggressive metastatic precursors than their epithelial counterparts, however the EpCAM-dependent CellSearch platform is unable to enrich these cells as EpCAM is downregulated either partially or completely during EMT [154,155]. Numerous systems have been developed to allow the enrichment of both epithelial and mesenchymal CTCs including filtration-based methods, size dependent microfluidic methods and dielectrophoretic methods, with the latter two groups proving to be more successful. Of the technologies reviewed herein, IsoFlux, Vortex and the ^LP^CTC-iChip are the three key approaches favoured for mesenchymal CTC enrichment. The ^LP^CTC-iChip may be of significant importance since the chip has been designed to efficiently process leukapheresis products where CTC numbers are ultimately greater due to the much larger volume of blood sampled. CTC analysis from leukapheresis products is likely to be extremely important when considering early-stage cancer patients who naturally have much fewer CTCs than patients with later-stage disease.

It is also important to note that enrichment approaches must provide a gentle sorting method for cells in order to preserve CTCs for subsequent downstream molecular analysis if this is required beyond CTC enumeration. As previously discussed, many downstream molecular assays require either a negligible leukocyte background or pure CTCs for precise molecular characterisation, so a secondary isolation step is often required following the primary enrichment step. Of the secondary isolation technologies available, the DEPArray currently offers superiority due to its automated DEP field sorting that allows users to recover single, pure CTCs from an enriched sample in a matter of hours.

Moving forward, systems that provide greater automation and higher throughput will be the most useful when considering routine clinical use, together with the flexibility of isolation of both epithelial and mesenchymal like CTCs. For routine clinical implementation, this will need to be balanced by affordability and ease of use of the platform and associated reagent costs.

## Figures and Tables

**Table 1:** CTC isolation technologies, grouped based on enrichment method. Capture efficiency, recovery rate and advantages and disadvantages of the technologies are also shown.

Subcategory	Name	Capture Efficiency (%)	Recovery Rate (%)	Advantages	Disadvantages
Immunomagnetic enrichment					
Immunomagnetic positive enrichment	CellSearch [3,4,5,6,7]	42–90		Semi automated Can process up to 8 samples at a time In device staining CTC enumeration via CellTracks Analyser FDA approved	Recovery of EpCAM^+^ CTCs only Only able to detect CTCs expressing high levels of EpCAM
	MACS [8,9]	25–90		Cocktail of antibodies available to increase CTC capture Able to process up to 15 mL blood Easy elution of CTCs Pro Separator can process up to 6 samples at once	Recovery of EpCAM^+^ CTCs only Suggested the MACS system is better suited for tissue samples
	MagSweeper [10,11]	60–70		Nonadherent plastic sleeves allow for multiple rounds of capture to increase capture efficiency	Recovery of EpCAM^+^ CTCs only
	Strep-tag [12,13]	79–86	70	Easy release of CTCs by simple addition of d-biotin Possibility to use a cocktail of antibodies to increase capture	Recovery of EpCAM^+^ CTCs only
	IMS [14]	92		Leukocytes repelled so high purity recoveries	Recovery of EpCAM^+^ CTCs only Not yet tested on patient samples
Immunomagnetic negative enrichment	EasySep [15,16]	19–65		Recovery of heterogeneous population of CTCs	Exclusion of CTC-WBC clusters Variable recovery rates May inadvertantly remove CTCs
	RosetteSep [17]	62.5		Recovery of heterogeneous population of CTCs Cocktail of antibodies used to maximise depletion	Exclusion of CTC-WBC clusters, May inadvertantly remove CTCs
Microfluidic immunocapture positive enrichment	CTC-Chip [18,19]		>60	Large surface area for CTC capture High viability of recovered cells	Recovery of EpCAM^+^ CTCs only Slow processing rate Complex geometry of chip difficult to scale up Geometry prevents passage of CTC clusters
	HB-chip [20]	74.5–97		HB grooves increase CTC-antibody contact for increased cell capture	Recovery of EpCAM^+^ CTCs only
	GEDI chip [21]	80–90		Large surface area for CTC capture Possibility to functionalise with alternative antibodies	May miss heterogeneity of CTCs
	HTMSU [22]	>97		Quick processing On-chip single-cell conductometric counting for enumeration	Recovery of EpCAM^+^ CTCs only
	Nanovelcro [23]	70–95		4 generations developed for different clinical utilities 3rd and 4th generation chips adapted for easy CTC release	Recovery of EpCAM^+^ CTCs only
	Isoflux [4]	74–90	64–75	Utilises microfluidic approach to increase EpCAM sensitivity Up to 4 samples can be processed in parallel Multiple kits including cocktails of antibodies to capture heterogeneity IsoFlux Cytation Imager for sample scanning	
Capture enhancement by nanomaterials	NP-^HB^CTC-Chip [24]	79–97		Simple release of CTCs by addition of glutathione (GSH) Chip surface can be functionalised with a cocktail of antibodies for enhanced capture efficiency	Recovery of EpCAM^+^ CTCs only Very low throughput
	GO chip [25,26]	67–100	91–95	Simple chip design Large surface area for increased CTC capture	Recovery of EpCAM^+^ CTCs only
	SiNP [27]	84–91		Large surface area for CTC capture	Recovery of EpCAM^+^ CTCs only
Capture enhancement by nanomaterials	Nanotube-CTC-chip [28]	89–100		Preferential adherence negates need for EpCAM antibodies Planar enrichment surface makes chip visualisation and imaging easy	Time taken for optimal CTC adherence to substrate is too long
Size based enrichment					
Membrane filtration	FMSA [29]	90		Recovery of heterogeneous population of CTCs Cheap and easy to produce Quick processing time	Filter clogging highly likely
	ScreenCell [30]		74–91	Recovery of heterogeneous population of CTCs Cheap and easy to produce Three different devices offered depending on downstream requirements Quick processing time	Unevenly distributed or fused pores can reduce capture efficiency
	ISET [31,32]		83–100	Recovery of heterogeneous population of CTCs Cheap and easy to produce Ability to process 12 samples in parallel	Slow processing time Blood must be diluted 1:10 to prevent membrane clogging
	SB microfilter [33]	78–83		Recovery of heterogeneous population of CTCs Cheap and easy to produce Quick processing time	Only 1 mL blood can be processed at a time due to device clogging
	FAST [34]		94–98	Recovery of heterogeneous population of CTCs Cheap and easy to produce Quick processing time	
Microfluidics	Parsortix [35]	42–70	54–69	Recovery of heterogeneous population of CTCs Ability to capture CTC clusters Option for on-chip staining	Slow processing time On-chip imaging difficult
	MCA [36]	>90	68–100	Recovery of heterogeneous population of CTCs Option for on-chip staining Ability to process up to 4 samples in parallel	
	ClearCell FX1 [37,38]		52–79	Recovery of heterogeneous population of CTCs Quick processing time No channel clogging observed	
	Vortex VTX-1 [39,40]		53.8–71.6	Recovery of heterogeneous population of CTCs Filters at channel inlet prevent channel clogging Fully automated process Quick processing time Associated BioView for enumeration Option to run in “high recovery” or “high purity” mode	
	p-MOFF [41]		91.6–93.75	Recovery of heterogeneous population of CTCs Quick processing time No channel clogging observed	RBC lysis and Ficoll density centrifugation required
Density based	OncoQuick [42,43]	25–87		Recovery of heterogeneous population of CTCs Up to 25 mL blood can be processed per tube	Low detection and recoveryrates
	AccuCyte [44]		81–90.5	Recovery of heterogeneous population of CTCs Allows for processing of multiple samples in parallel Associated CyteFinder and CytePicker systems for imaging and mechanical selection of CTCs	
Other					
Dielectrophoresis	ApoStream [45,46]		55–78.5	Recovery of heterogeneous population of CTCs Quick processing time iCys laser scanning cytometer for enumeration High viability of recovered cells	
In vivo	Diagnostic leukapheresis (DLA) [47]			Recovery of heterogeneous population of CTCs Recovery of much greater numbers of CTCs	Only a pre-enrichment step so must be used in combination with another enrichment technology Huge leukocyte background
	GILUPI CellCollector [48]			Potential for much greater numbers recovered	More invasive for the patient than a simple blood draw Recovery of EpCAM^+^ CTCs only
Combined	CTC-iChip [49]		70–100	Option for positive or negative enrichment approach Inertial focusing provides high sensitivity selection Quick processing time	Positive enrichment only allows for recovery of EpCAM^+^ CTCs Negative enrichment will exclude CTC-WBC clusters
	^LP^CTC-iChip [50]		85.5–100	Potential for much greater numbers recovered Magnetic field directs WBCs to centre of channel to prevent channel clogging Extremely high throughput	Disregards CTC-WBC clusters Initial debulking step may result in CTC loss
	OPENchip [51]		50	Chip allows for CTC enrichment and on-chip downstream molecular analysis	Low throughput, low recovery rates

## Data Availability

Not applicable.
